# Regulation of the Human Papillomavirus Life Cycle by DNA Damage Repair Pathways and Epigenetic Factors

**DOI:** 10.3390/v12070744

**Published:** 2020-07-10

**Authors:** Ekaterina Albert, Laimonis Laimins

**Affiliations:** Department of Microbiology-Immunology, Northwestern University, Feinberg School of Medicine, Chicago, IL 60611, USA; ekaterina.albert@northwestern.edu

**Keywords:** HPV, DNA damage, ATM, ATR, sirtuins, Tip60

## Abstract

Human papillomaviruses are the causative agents of cervical and other anogenital cancers along with approximately 60% of oropharyngeal cancers. These small double-stranded DNA viruses infect stratified epithelia and link their productive life cycles to differentiation. HPV proteins target cellular factors, such as those involved in DNA damage repair, as well as epigenetic control of host and viral transcription to regulate the productive life cycle. HPVs constitutively activate the ATM and ATR DNA repair pathways and preferentially recruit these proteins to viral genomes to facilitate productive viral replication. In addition, the sirtuin deacetylases along with histone acetyltransferases, including Tip60, are targeted in HPV infections to regulate viral transcription and replication. These pathways provide potential targets for drug therapy to treat HPV-induced disease.

## 1. Introduction

Human papillomaviruses are small, double-stranded DNA viruses approximately 8 kb in length that are the etiological agents of cervical cancers, as well as many other malignancies of the anogenital tract. Over 400 human papillomaviruses (HPV) types have been identified, of which about 200 have been approved by the International Committee on Taxonomy of Viruses (ICTV) to date. Only a small subset of these is associated with human cancers [1]. A group of approximately 10 types from the alpha genus are referred to as high-risk and are the causative agents of virally induced anogenital cancers [2]. The most prominent of this group are HPV 16, 18, 31, 33, 35, and 45. HPV 16 is associated with approximately 50% of cervical cancers, while 25% are linked with HPV 18, and the rest with the remaining 25% [2]. Interestingly, high-risk HPVs are also associated with approximately 60% of cancers of the oropharynx, and this is primarily attributed to HPV 16 [3,4].

Up to 1/3 of all HPV types certified by the ICTV infect the genital tract and are sexually transmitted. Approximately 2/3 of young adults acquire HPV infections in the genital tract in the first few years of sexual activity [5,6]. Most of these infections are cleared by the immune system within a year or two; however, a small percentage of women fail to clear infections by the high-risk types and become persistently infected. These persistently infected women are at elevated risk for developing cervical cancer within decades of initial infections. Cervical cancer is the fourth most common cancer among women worldwide, and these malignancies are more frequently seen in developing countries [7]. The number of cases in Western countries has decreased dramatically in the past few decades with the use of the Pap smear diagnostic, which can identify premalignant lesions before they progress to cancer, allowing for therapeutic intervention. The lack of effective screening in developing countries is likely responsible for the high incidence of HPV-induced disease worldwide.

**Figure 1:**
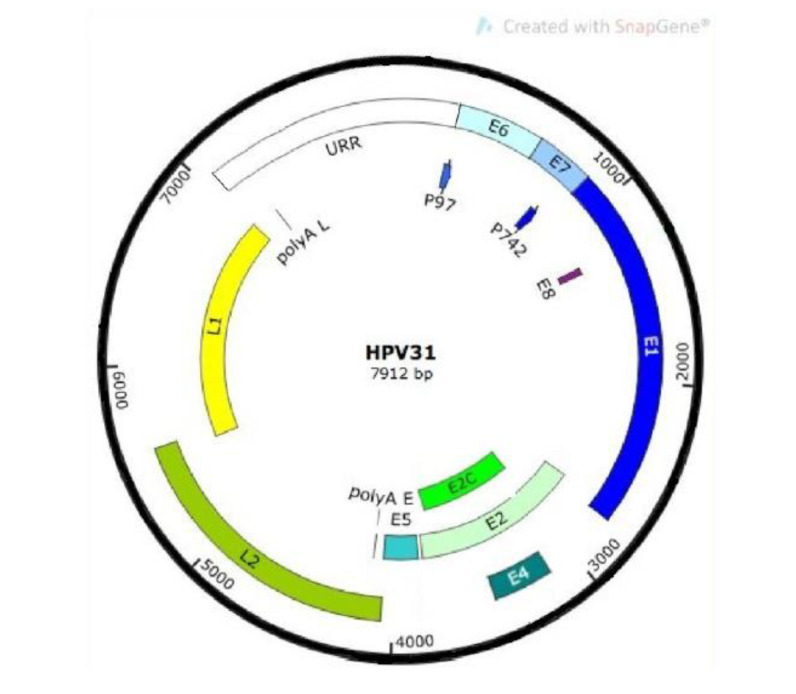
HPV 31 genome map showing the upstream regulatory region (URR), early genes E1, E2, E4, and E5, as well as late capsid genes L1 and L2. The early promoter is designated p97, while the late differentiation-dependent promoter is p742. Early and late poly A sites are shown.

HPVs infect cells in the basal layer of stratified epithelia and establish their genomes as low-copy nuclear episomes. Productive replication and assembly of progeny virions only occurs upon differentiation. HPV genomes can be subdivided into three functional regions: the upstream regulatory region (URR), the early (E) region, and the late (L) region (Figure 1).

The URR region does not code for any proteins and encompasses almost an eighth of the entire HPV genome. It contains the viral origin of replication (ORI) and the early promoter regulatory elements. The other two regions encode approximately eight open reading frames (ORF) that are expressed from polycistronic mRNAs. The E region encodes for the HPV early genes E1, E2, E4, E5, E6, and E7. These early genes have critical functions in the viral life cycle, including HPV genome replication, gene expression, immune system evasion, and viral genome persistence [8]. The two genes encoded by the L region consist of the structural proteins L1 (major capsid protein) and L2 (minor capsid protein) that are produced late in the viral life cycle and form the viral capsid [8]. The products of the HR-HPV E6 and E7 genes are the two major viral transforming proteins. The sustained expression of these two proteins is required to induce and maintain the oncogenic properties of transformed host keratinocytes [9,10]. Specifically, the E6 and E7 oncogenes can independently immortalize primary epithelial cells by targeting cellular proteins that control the mitotic checkpoint and the p53-directed cell cycle arrest that is normally triggered by DNA damage [9].

A major target of the E6 protein is the tumor suppressor protein, p53. E6 forms a ternary complex with the E6-associated protein (E6AP) ubiquitin ligase and p53, promoting its degradation by ubiquitin-mediated proteolysis [11,12]. The E7 protein binds and promotes the degradation of members of the retinoblastoma (pRb) tumor suppressor proteins, pRb1^p110^, p107 and p130 (pRb2), with functions in cell cycle control and transcription [13,14,15]. E6 and E7 can also bind a number of other cellular factors, including PDZ proteins and p600, which can augment transforming activities [16,17].

The E1 and E2 proteins are the two virally encoded replication factors that bind to origin sequences located near the start of early transcription. Three E2 binding sites flank a single E1 binding sequence, and complex formation between E1 and E2 facilitates recruitment to viral origins [18,19]. E2 also regulates viral transcription, while E1 acts as a helicase and also recruits cellular replication factors to the viral origin. A truncated E2 protein called E8^E2C acts as a repressor of viral transcription and helps modulate levels of expression throughout the life cycle [20]. The E1^E4 protein is the most highly expressed of all viral proteins, and is thought to facilitate virion egress following assembly in suprabasal cells [21]. The E5 protein localizes to membranes and may regulate epidermal growth factor receptors (EGFR) signaling in differentiating cells [22,23].

## 2. Human Papillomavirus Life Cycle

**Figure 2:**
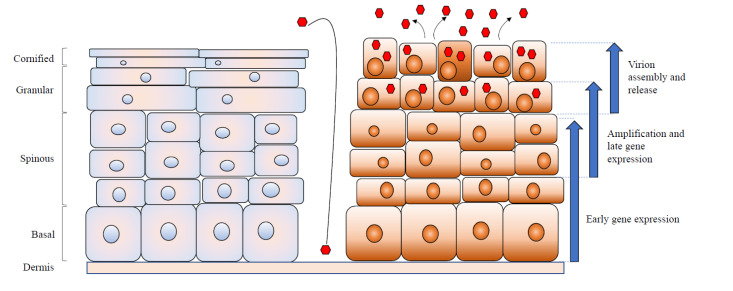
HPV life cycle. Normal keratinocytes are shown on the left, while HPV-infected keratinocytes are shown on the right. HPV virions infect basal cells that become exposed upon wounding. Following entry, low-copy episomes are established in undifferentiated cells and are transmitted to daughter cells, one of which goes on to differentiate. Upon differentiation, genome amplification is induced, along with late promoter activation. This is followed by virion assembly and release.

Initial infection by HPV virions occurs in basal epithelial cells that become exposed following wounding (Figure 2). Following entry, HPV genomes migrate to the nucleus in vesicles together with the L2 protein, where they are established as low-copy episomes [24]. Once in the host cell nucleus, HPV genomes quickly replicate up to 10–200 copies/cell, marking an initial amplification phase and resulting in the establishment of infection [25]. During this initial phase, only the early viral promoter is transcriptionally active, leading to the expression of HPV early proteins, including E1 and E2, as well as E6 and E7 [25].

HPV genomes in infected basal cells replicate in synchrony with chromosomal DNA replication in the S phase, and the resulting newly replicated genomes are distributed equally to the two daughter cells [8]. One daughter cell remains in the basal layer to continue to proliferate, while the other daughter cells migrate toward suprabasal layers and undergo differentiation. As the HPV-positive cells undergo differentiation, viral genome replication switches to a productive mode concomitant with increased levels of E1 and E2 expression, resulting in the synthesis of thousands of genome copies [25]. In the terminally differentiated layer of the epithelium, the L1 and L2 capsid proteins are expressed under the control of the late promoter, and assembly of the mature viral particle takes place. Virions are shed together with the dead squamous cells of the exterior-most epithelium for further transmission [25].

## 3. High-Risk Human Papillomavirus Integration into the Genome of the Host Keratinocyte

In precancerous lesions harboring HR-HPV, the genomes are present as extrachromosomal episomes. In contrast, in cervical squamous cell carcinomas, HR-HPV DNA is frequently found integrated into the human genome [8]. In general, there is a correlation between progression to cancer and the physical state of the viral genome, with episomes being predominant in early stages, while integrated viral genomes are more frequently detected in high-grade lesions and carcinomas [26]. HR-HPV integration is considered to be an early event in carcinogenesis, and is thought to be mechanistically linked to virus-induced malignancy [27]. Integration of viral genomes frequently occurs in the E2 ORF so as to disrupt its expression, resulting in increased expression of E6 and E7 that further contributes to malignant progression [8]

## 4. Modes of HPV Genome Replication

During the productive life cycle in stratified epithelia, HPV genomes undergo three distinct modes of replication [28,29]. During the first phase, shortly after initial entry into the nucleus, there is a rapid increase in viral genome DNA replication to increase the stable episome copy number. This phase is dependent on the viral replicative E1 and E2 proteins, which recruit and direct the cellular replication machinery to the viral origin within the URR.

In the second phase of viral genome replication, the so-called stable maintenance phase, a constant copy number of viral genomes is maintained through bidirectional theta replication of viral episomes that occurs in synchrony with chromosomal replication [30]. This mode of viral replication is characterized by circular molecules containing bi-directional replication forks, which initiate at the ORI and proceed in opposite directions, and is again dependent on the viral E1 and E2 proteins.

The third phase of vegetative amplification is characterized by the rapid production of high numbers of viral genomes, where evidence has been seen for both bidirectional theta replication and recombination-dependent replication (RDR; [29]). RDR is a viral replication mechanism that lacks specific ORI sequences and that can initiate replication within various regions of the HPV genome. In contrast to bidirectional theta replication, RDR has a unidirectional replication fork [29,31]. Viral DNA and its replication intermediates are also sensed as damaged DNA by the host cell, which thus initiates a DNA damage response that is utilized by the virus for its amplification program which occurs in G2/M, rather than the S phase [32]. Expression of E6, E7, or E1 by themselves can activate these pathways by inducing replication stress [29,33].

## 5. The DNA Damage Response

To maintain genomic integrity, cellular DNA must be protected from breaks induced by environmental agents, or generated spontaneously during normal cellular metabolism. The DNA Damage Repair pathways (DDR) are comprised of several complex, sometimes overlapping, cellular mechanisms, sensing damage incurred to DNA, transducing this information, and eliciting a variety of cellular responses [34]. These responses include undergoing apoptosis, entering terminal differentiation through senescence, activation of immune surveillance, as well as repair of damaged DNA [35,36]. To counteract DNA damage, repair mechanisms specific to many types of lesions can be activated. Two major types of DNA lesions are single-strand breaks (SSB) and double-strand breaks (DSB). SSB are repaired by single-strand break repair (SSBR), while the two main pathways of DSB repair are either non-homologous end joining (NHEJ) or a homologous recombination (HR) [34,36].

**Figure 3:**
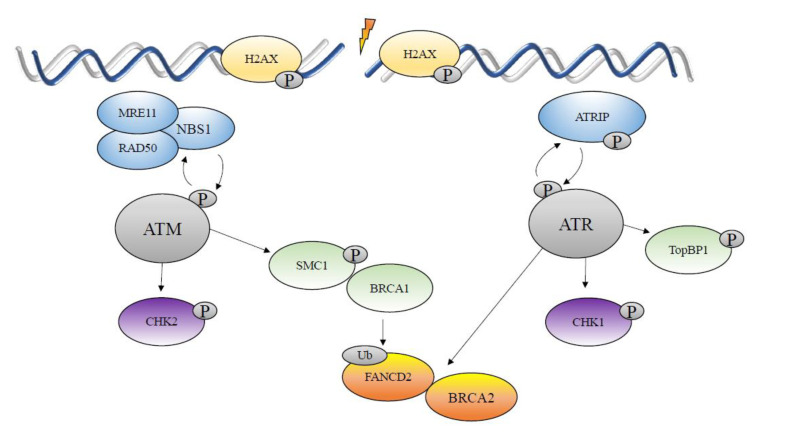
ATM and ATR DNA damage repair pathways. Upon DNA damage, the MRE11-RAD50-NBS1 (MRN) complex recruits ATM to the site where it undergoes autophosphorylation. The activated ATM kinase then phosphorylates the histone H2AX, CHK2, SMC1, and other downstream effectors. At the same time, ATR is activated by phosphorylation through association with TOPBP1 and ATRIP, leading to phosphorylation of CHK1, H2AX, TOPBP1, and ATRIP. This leads to phosphorylation of hundreds of downstream factors, resulting in homologous recombination repair in G2/M.

The central, often termed “apical”, players of the DDR comprise proteins of the phosphatidylinositol 3-kinase-like protein kinase (PIKK) family—ataxia telangiectasia mutated (ATM), ataxia telangiectasia and Rad3-related (ATR) (Figure 3), and DNA-dependent protein kinase catalytic subunit (DNA-PKcs), as well as members of the poly(ADP)ribose polymerase (PARP) family [34]. The ATM- and DNA-PK-dependent repair pathways are activated by DNA-damaging agents that create DSB, such as ionizing radiation [36,37]. The ATR-dependent pathway is activated by long stretches of single-stranded DNA (ssDNA) formed, for example, by stalled replication forks [38]. In fact, ATR is the principal mammalian cell player involved in the tolerance of replication stress-induced DNA damage [38].

ATM is recruited to sites of double-strand breaks by the MRN (MRE11- RAD50-NBS1) complex, which results in its activation by autophosphorylation and acetylation by the acetyltransferase Tip 60. The NBS1 component of the MRN complex has been shown to associate with ATM via its C-terminal region, promoting the recruitment of ATM to DSB. Activated ATM then induces phosphorylation of the specialized histone H2AX at Ser139, forming γH2AX [39]. Phosphorylation of histone H2AX occurs specifically and rapidly in response to DSB, and is catalyzed by the PIKK family of kinases themselves [39,40]. H2AX phosphorylation can spread for up to 1–2 mega-bases around the DSB, thus effectively amplifying the DNA damage signal to the cell and its DDR machinery [41].

Once activated by auto-phosphorylation, ATM phosphorylates more than 700 downstream targets in addition to histone H2AX [42]. These include the checkpoint kinase 2 (CHK2), involved in cell cycle progression and apoptosis, the 53BP1 protein functioning in DSB repair and checkpoint maintenance, the NBS1 component of the MRN (MRE11-RAD50-NBS1) complex, involved in cellular checkpoints and DNA repair, and the tumor suppressor Breast Cancer 1 (BRCA1) with critical functions in cell cycle control and the DDR [42].

ATR is activated by replication fork stalling and formation of extensive single-strand DNA regions that are coated with RPA proteins. ATR in complex with its binding partners ATRIP, TOPBP1, and claspin is recruited to these sites, leading to its activation. Downstream targets of ATR include the checkpoint kinase 1 (CHK1), which is structurally distinct from CHK2 but has overlapping roles in cell cycle regulation and apoptosis, the Breast Cancer 2 (BRCA2) tumor suppressor, promoting RAD51 catalytic activity and recruitment to DSB sites during, H.R.; and FANCD2, the central executor protein in the repair of DNA cross-links by the Fanconi Anemia (FA) pathway [42]. There is a significant overlap between ATM and ATR phosphorylation substrate specificity [42].

ATM, ATR, along with their downstream effector kinases CHK2 and CHK1, respectively, all phosphorylate the tumor suppressor protein p53 [43,44,45]. Phosphorylation of p53 interferes with its ubiquitination by the E3 ubiquitin-protein ligase MDM2, thus inhibiting degradation of p53 by the proteasome [46].

Phosphorylation of the effector proteins CHK2 or CHK1 kinase also leads to the phosphorylation of a large number of cellular substrates. This results in cell cycle arrest at the G2/M transition, as part of the DNA damage checkpoint, allowing for the repair of DNA lesions before cell division [36]. Homologous recombination occurs in G2/M as it requires a sister chromatid template.

## 6. Double-Strand Break Repair

DSB are the most toxic DNA lesions and their repair is promoted by an intricate network of multiple DNA repair pathways. The two main DSBR pathways that have evolved in the eukaryotic cell are the non-homologous end joining (NHEJ) and homologous recombination (HR). There is very little evidence for involvement of NHEJ in HPV biology, while multiple studies point to a role for HR in the viral life cycle. Therefore, discussion will be limited to this latter pathway of DSBR.

## 7. Homologous Recombination Repair

During DSB repair by, HR; PARP1 is thought to assist in the initial accumulation of the MRN complex at DSB sites [47]. Recruitment of ATM by MRN and PARP1 contributes to the HR cascade and the stabilization of DDR factors at DNA damage sites [47]. The MRE11 component of the MRN complex also exhibits endo- and exonuclease activities, and participates in the initial steps of broken DNA end resection, essential for the HR process. In addition to its many other functions in HR, ATM also regulates the DNA end resection at the DSB site through activation by phosphorylation of the nuclease CtIP, which also interacts with the MRN complex. CtIP nuclease activity is enhanced by its interaction with the Breast Cancer 1 protein (BRCA1), which in turn is activated by ATM-mediated phosphorylation [48].

DSB resection leads to the formation of single-stranded (ss) DNA, which is quickly coated by the heterotrimeric complex replication protein A (RPA), stabilizing ssDNA regions [49]. Subsequent competitive displacement of RPA molecules by RAD51 and assembly of RAD51 filaments onto ssDNA is mediated and enhanced by the BRCA2 protein [50,51]. RAD51 filament formation promotes, and is essential for, HR [52]. Following RAD51-catalyzed strand invasion into homologous sequences of the sister chromatid and the formation of a “D-loop”, the 3′-invading and 5′-non-invading DNA strands are extended by specialized DNA polymerases, and the invading strand finally reanneals to the processed second end of the break, completing repair [52]. The extensive DNA resection associated with HR is primarily induced in the S and G2 phases of the cell cycle, when sister chromatids are available to be used as templates for HR-mediated repair [53].

The Structural Maintenance of Chromatin (SMC) proteins, originally identified in yeast as promoters of the proper condensation and segregation of mitotic chromosomes [54], have also been pinpointed as players in the ATM-mediated HR DNA repair response. In particular, the Smc1 component of the evolutionarily-conserved SMC1-SMC3 heterodimer, involved in sister chromatid cohesion, is phosphorylated in an ATM-dependent manner following exposure to irradiation [55]. Phosphorylation of SMC1 is required for the ATM-mediated checkpoint responses and the HR process [55].

## 8. Stalled DNA Replication Forks

Bulky DNA lesions (such as chemically induced modified bases or inter-strand DNA cross-links) can lead to replication fork stalling, uncoupling between the replicative helicase and the DNA polymerase, and the resulting formation of extensive RPA-coated ssDNA regions [56]. These RPA-ssDNA regions are potent activators of the ATR pathway, which is the central DDR pathway involved in the management of DNA replication stress [56]. Specifically, RPA-coated ssDNA directly recruits ATR complexed with its binding partner, ATR Interacting Protein (ATRIP), localizing them to stalled forks [36]. This fires off the ATR signaling cascade, which leads to the activation of the CHK1 effector kinase and the phosphorylation of a multitude of chromatin-bound factors, as well as non-histone proteins [36]. Collectively, this promotes fork stability and the restart of stalled or collapsed replication forks, so that chromosome replication can be completed [38]. Amongst others, restart of stalled replication forks is dependent on the Fanconi Anemia protein FANCM. ATR-dependent phosphorylation of FANCM in response to genotoxic stress has been shown to be essential for the recruitment of FANCM to inter-strand crosslinks and efficient activation of the CHK1-induced G2/M checkpoint, preventing cells from entering mitosis prematurely [57].

## 9. DNA Interstrand Cross-Links and the FANC Group of Proteins

The protein products of the Fanconi Anaemia (FA) group of genes are involved in the resolution of a specialized type of DNA damage—DNA interstrand cross-links (ICL). These lesions, resulting from the covalent cross-linking between bases on two opposite DNA strands, can be particularly deleterious, as they can impede the progression of DNA replication forks and cause their stalling and collapse. There are currently 22 known FA genes in the complementation group, and they are designated FANCA through FANCW [58]. The stalled fork at the ICL is first recognized and signaled by FANCM, in concert with other cellular factors. In particular, FANCM functions to promote the ATR kinase-mediated checkpoint response. As a consequence, the FA core complex (comprising the majority of proteins in the FA group) mono-ubiquitinates the FANCI-FANCD2 dimeric complex, which primes ubiquitinated FANCD2, together with other proteins, to perform the “unhooking” step in the resolution of the ICL in which a DSB is created on the DNA strand opposite the strand harboring the cross-linked nucleotide. DNA replication can resume through lesion bypass, which is achieved through the action of specialized translesion polymerases. The DSB ends can be processed as in a typical HR event, generating ssDNA stretches through classical DSB resection. The FA pathway therefore overlaps significantly with the HR pathway and is, not surprisingly, sometimes called the FA/HR pathway.

## 10. DNA Damage Repair Pathways and HPV replication

The ATM and ATR DNA damage repair pathways are constitutively activated in high-risk HPV-positive cells in the absence of any DNA damaging agents [8,59,60]. The downstream phosphorylation targets of the pATM and pATR kinases are also activated in these cells. For the ATM pathway, this includes pCHK2, γH2AX, pBRCA1, and pNBS1. Following DNA damage in normal cells, members of the ATM and ATR pathways are activated and then co-localize to multiple nuclear foci that can be readily seen by immunofluoresence [61]. In HPV-positive cells, similar localizations of ATM and ATR factors to nuclear foci are observed. Importantly, these foci are also sites of HPV replication, and the corresponding DNA damage repair factors are found to be bound to viral genomes. Treatment of cells with HPV episomes either with inhibitors of ATM or knockdown of CHK2, RAD51, BRCA1, as well as NBS1 leads to an inhibition of differentiation-dependent viral genome amplification, but with minimal effect on stable maintenance replication in undifferentiated cells [28,61,62]. In contrast, inhibition of the ATR pathway prevents both differentiation-dependent amplification, as well as stable maintenance replication in undifferentiated cells [60,63]. These two DNA damage repair pathways are thus hijacked in HPV-positive cells where they are required for viral genome replication. Interestingly, while levels of activated ATM factors are seen in cells expressing E6 and E7, the activities of these proteins are partially impaired in double-strand break repair, which may contribute to viral genome integration and which is often seen in HPV-positive cancers [64].

In HPV-positive cells, the ATM and ATR pathways are activated by the action of viral proteins alone and do not require viral replication, though this can augment the level of activation. The expression of either E6 or E7 proteins alone is sufficient to activate both ATM and ATR pathways (Table 1) [33,65].

The E7 protein acts in part through activation of the innate immune transcriptional regulator, STAT-5, which increases expression of TOPBP1, an ATR activator, and also activates ATM [65]. Activation of ATM and ATR also correlates with the induction of enhanced levels of DNA breaks as compared to cells not expressing viral oncoproteins. Interestingly, in cells with viral episomes, DNA breaks are induced in both viral and cellular DNAs, but as a result of enhanced recruitment of DNA damage repair factors to HPV genomes, leading to preferential repair and amplification [33]. Several mechanisms have been suggested as responsible for the enhanced induction of DNA breaks by E6 and E7. The interaction with the RB family of proteins results in an increase in the frequency in initiation of DNA replication from cellular origins, which then results in many stalled replication forks, due to either depleted nucleotide pools or formation of R-loops from collisions of rapidly induced RNA and DNA polymerases. Recent work has, however, shown that HPV 31 increases nucleotide pools through enhanced levels of the RRM2 ribonucleotide reductase complexes [66]. Alternatively, interactions of viral proteins with cellular replication factors may also contribute. For instance, E7 has been shown to bind to the E3 ubiquitin ligase, RNF168, which is critical for proper DNA repair leading to enhanced viral replication [67]. In addition to E6 and E7, high-level expression of E1 from heterologous promoters has also been shown to activate the ATM DNA damage response. This is suggested to be the result of spurious induction of replication from cellular origins resembling E1 binding sites, leading to onion skin-type structures and stalled replication forks. Similar effects could be seen with E1 mutants that were unable to shuttle from the nucleus and induced an ATM DNA damage response [68]. Whether wild-type E1 significantly activates the DNA damage response when expressed at low levels from viral promoters remains to be tested.

The cohesin protein SMC-1 can also be phosphorylated by ATM and plays a critical role in the homologous recombination repair process that requires a sister chromatid template. SMC1 is constitutively phosphorylated in both undifferentiated, as well as differentiated HPV-positive cells [33,69], and co-localizes to viral replication foci along with other homologous DNA repair proteins. pSMC-1 proteins bind to HPV genomes in complexes with the CTCF insulator proteins that are bound to conserved CTCF sites on viral DNAs. Importantly, knockdown of CTCF proteins or SMC-1 completely inhibits viral productive replication upon differentiation. All of the above described DNA repair factors are in the homologous recombination repair pathway that occurs in G2/M, which is also when HPV genome amplification takes place.

The Fanconi anemia (FA) pathway also provides critical functions for DNA repair. The FA pathway repairs interstrand cross-linked DNAs and is regulated by both the ATM and ATR pathways. ATR phosphorylates several proteins in FA pathway and this is required for optimal FANCD2 monoubiquitination and activation. ATM can also phosphorylate FANCD2, but this leads to an S-phase arrest [70]. FA patients have an increased incidence of HPV-positive head and neck cancer as well as cervical cancer implicating this pathway as critical for HPV pathogenesis [71]. In E7 expressing transgenic mice knockout of FANCD2 resulted in a propensity towards head and neck carcinomas [72].

FA proteins are intimately linked with the HPV life cycle and the development of HPV-related neoplastic disease. For example, loss of FANCA and FANCD2 leads to the accumulation of the high-risk E7 proteins in keratinocytes [73]. This in turn results in epithelial proliferation and basal cell-layer expansion in HPV-positive lesions, which may affect amplification [73]. In cells with HPV episomes, increased levels of FANCD2 proteins are observed, and these are preferentially recruited to nuclear repair foci. FANCD2 is preferentially recruited to viral episomes as compared to cellular sequences, and is required for stable maintenance replication [74]. Additional work has shown that while E6 and E7 can increase FANCD2 levels and foci formation, FANCD2 function is actually impaired by E6, leading to increased ATR activation [75]. How FANCD2 and other FA proteins contribute to HPV pathogenesis is an important area for future study.

## 11. Acetylation by Tip60

Activation of ATM in response to DNA damage is correlated not only with rapid autophosphorylation, but also its acetylation [76]. The responsible acetylase is the histone acetyl-transferase (HAT) Tip60, and acetylation is required for its autophosphorylation. Tip60 mutant cells exhibit enhanced sensitivity to ionizing radiation due to impaired activation of ATM and phosphorylation of key substrates, such as p53 and CHK2. Tip60 also acetylates chromatin [76].

Tip60 levels are increased in HPV-positive keratinocytes that maintain complete viral genomes [77]. In contrast, keratinocytes expressing only E6 and/or E7 show a decreased Tip60 level, suggesting that other viral proteins, such as E2, may contribute to its activation. In cells carrying complete HPV genomes, knock-down of Tip60 with shRNA inhibits HPV genome amplification [77]. This is not surprising, given the role of Tip60 in activating ATM for phosphorylation of itself and downstream targets [76]. Tip60 activation depends on STAT5, as inhibition of STAT5 with pimozide results in a dramatic decrease in Tip60 levels [77]. Tip60 also regulates chromatin remodeling and transcription by acetylating histones. Tip60 regulates expression of a number of transcription factors, such as the E2Fs and c-Myc, which have important roles in regulating HPV pathogenesis [76].

## 12. Sirtuin Deacetyalses

Sirtuins are a family of lysine deacetylases with homology to the budding yeast, silent information regulator 2 (Sir2), which regulates the formation of heterochromatin through the deacetylation of lysine residues on histone tails [78]. The human sirtuin family contains seven members: SIRT1 through SIRT7. While SIRT 6 and 7 predominantly show nuclear localization, SIRT3 4 and 5 are mitochondrial, and SIRT2 is primarily cytosolic [79]. Similar to the prototypical Sir2, human sirtuins are histone deacetylases that require NAD+ for their activity [80]. Through their effects on the acetylation of histones, sirtuins are involved in the regulation of gene expression, but also function in the deacylation of long-chain fatty acids and mono-ADP ribosylation. Each sirtuin has hundreds of non-histone substrates, and so regulates a number of cellular processes, such as control of energy metabolism, cell survival, DNA repair, and tissue regeneration [80].

SIRT1 is the most extensively studied member of the sirtuin family and deacetylates histones H3, H4, and H1 [81]. It has more than 750 additional non-histone targets, including transcription factors such as p53 and NF-kB [82]. In response to DNA damage, SIRT1 regulates both chromatin compaction and DDR activity. SIRT1 can relocate from transcriptionally repressed repetitive DNA loci to DNA breaks to promote repair of damaged DNA by the ATM signaling pathway [83]. SIRT1-promoted repressive histone modifications maintain chromatin compaction around the DNA damage site so as to protect the broken DNA duplex during repair [83]. In addition, SIRT1 can recruit repair proteins, including γH2AX, Rad51, BRCA1, and Nbs1, to DSB sites and modulate their activity via deacetylation [84], which makes it a regulator of both DNA repair and the establishment of cell cycle checkpoints in response to DNA damage. Finally, binding and deacetylation of Nbs1 by SIRT1 directly promotes phosphorylation of Nbs1 by ATM upon exposure to ionizing radiation [83].

SIRT6 also has a multitude of reported functions in DNA damage repair, promoting resistance to DNA damage and suppressing genomic instability. SIRT6-deficient cells display marked genomic instability and are hypersensitive to DNA-damaging agents, such as ionizing radiation, methylmethane sulfonate, and hydrogen peroxide [85]. Like other sirtuins, SIRT6 is a NAD+-dependent deacetylase, with histones H3K9 and H3K56 as substrates [86]. It regulates telomeric chromatin, gene expression, and the dynamic binding of DNA repair factors to chromatin [85]. Notably, SIRT6 renders more efficient the two kinds of DSB repair in the cell, HR and NHEJ. SIRT6 also interacts with and ADP-ribosylates PARP1, stimulating its catalytic activity within the DDR process [87]. In addition, SIRT6 is responsible for the recruitment of downstream DNA repair factors within the DDR cascade, both in vitro and in vivo [85].

SIRT2 is the only Sirtuin found in the cytoplasm during most of the cell cycle, but translocates into the nucleus during mitosis [88]. SIRT2 ensures the proper maintenance of the mitotic checkpoint by modulating the activity of the anaphase-promoting complex/cyclosome (APC/C). It performs this function through the deacetylation of two co-activators of the APC/C: CDH1 and CDC20. In addition, SIRT2 functions in the maintenance of genome stability and integrity in response to replication stress [89]. First, it deacetylates CDK9, which is required for efficient recovery from replication arrest following replication fork stalling. SIRT2 also deacetylates ATR-interacting proteins (ATRIP), leading to ATR activation via auto-phosphorylation. This activation of ATR facilitates recovery from replication stress, ultimately serving to maintain genome integrity [89].

## 13. SIRT1 and HPV

SIRT1 is the only Sirtuin that has been studied in relation to HPV biology. SIRT1 levels are increased in HPV-positive cells through the combined action of the E6 and E7 oncoproteins [90]. Knock-down of SIRT1 with shRNA dramatically impairs key viral activities, such as genome maintenance, amplification, and late gene transcription [90]. In undifferentiated cells, SIRT1 binds multiple regions of the viral genome, but this association is decreased upon cellular differentiation. The histone targets of SIRT1 deacetylation within the HPV genome are H4K26 and H4K16, which may be responsible for SIRT1 effects on productive replication and late gene expression [90]. In addition, SIRT1 modulates the recruitment of HR factors to the viral genome, including NBS1 and Rad51, which may play an additional role in the viral replication cycle. It has also been reported that SIRT1 binds to the viral replication complex consisting of proteins E1 and E2, and can positively regulate HPV16 E1- and E2-mediated viral DNA replication during initial infections [91]. While the levels of SIRT2 and SIRT6 are increased in HPV-positive cells, their contributions to HPV pathogenesis have yet to be fully elucidated.

## 14. Summary

HPV proteins activate a number of cellular pathways to facilitate viral replication. This includes those involved in DNA damage repair, as well as epigenetic control of host and viral transcription. HPVs constitutively activate the ATM and ATR DNA repair pathways and preferentially recruit these proteins to viral genomes. Both these pathways are required for productive viral replication. Epigenetic modifiers that regulate acetylation also play important roles. The sirtuin deacetylases, along with histone acetyltransferases including Tip60 are required in HPV infections to regulate viral transcription and replication. These pathways are also important as they could be useful targets for drug therapy to treat HPV-induced disease.

## Figures and Tables

**Table 1:** High-risk HPV E6 and E7 proteins increase the levels of total and phosphorylated forms of DNA damage repair proteins. A list of DNA damage repair proteins whose levels are increased in HPV-positive cells, as well as those that become phosphorylated through the action of E6 and E7.

DDR proteins affected by E6 and E7 Header	Proteins
A. Increased total levels of DDR proteins	ATR
	CHK1
	TOPBP1
	FANCD2
	RAD51
	NBS1
	BRCA1
	53BP1
	RNF168
	Tip60
B. Increased levels of phosphorylated forms	pATM
	pCHK2
	pNBS1
	pBRCA1
	γH2AX
	pATR
	pCHK1
	pTOPBP1
	pSMC1
	pMRE11
